# Nest expansion assay: a cancer systems biology approach to *in vitro *invasion measurements

**DOI:** 10.1186/1756-0500-2-130

**Published:** 2009-07-13

**Authors:** Yoonseok Kam, Audrey Karperien, Brandy Weidow, Lourdes Estrada, Alexander R Anderson, Vito Quaranta

**Affiliations:** 1 Integrated Mathematical Oncology, Moffitt Cancer Center, Tampa, FL, USA; 2 School of Community Health, Charles Sturt University, Albury, Australia; 3 Department of Cancer Biology, Vanderbilt University School of Medicine, Nashville, TN, USA

## Abstract

**Background:**

Traditional *in vitro *cell invasion assays focus on measuring one cell parameter at a time and are often less than ideal in terms of reproducibility and quantification. Further, many techniques are not suitable for quantifying the advancing margin of collectively migrating cells, arguably the most important area of activity during tumor invasion. We have developed and applied a highly quantitative, standardized, reproducible Nest Expansion Assay (NEA) to measure cancer cell invasion *in vitro*, which builds upon established wound-healing techniques. This assay involves creating uniform circular "nests" of cells within a monolayer of cells using a stabilized, silicone-tipped drill press, and quantifying the margin expansion into an overlaid extracellular matrix (ECM)-like component using computer-assisted applications.

**Findings:**

The NEA was applied to two human-derived breast cell lines, MCF10A and MCF10A-CA1d, which exhibit opposite degrees of tumorigenicity and invasion *in vivo*. Assays were performed to incorporate various microenvironmental conditions, in order to test their influence on cell behavior and measures. Two types of computer-driven image analysis were performed using Java's freely available *ImageJ *software and its *FracLac *plugin to capture nest expansion and fractal dimension, respectively – which are both taken as indicators of invasiveness. Both analyses confirmed that the NEA is highly reproducible, and that the ECM component is key in defining invasive cell behavior. Interestingly, both analyses also detected significant differences between non-invasive and invasive cell lines, across various microenvironments, and over time.

**Conclusion:**

The spatial nature of the NEA makes its outcome susceptible to the global influence of many cellular parameters at once (e.g., motility, protease secretion, cell-cell adhesion). We propose the NEA as a mid-throughput technique for screening and simultaneous examination of factors contributing to cancer cell invasion, particularly suitable for parameterizing and validating Cancer Systems Biology approaches such as mathematical modeling.

## Background

Classical wound-healing, cell migration, and cancer invasion assays have been carried out in tissue culture for decades, primarily to generate information about the relationship between cell motility and invasion [1-3]. However, a number of these techniques are encumbered with problems of quantification, reproducibility, and flexibility. For example, traditional wound-healing, or "scratch" assays include creation of an artificial wound (i.e., a scratch) within a monolayer of cells using a blunt object (e.g., pipet tip), and subsequent quantification of cells repopulating the scratch over time [1]. Not surprisingly, such assays often produce crude quantitative data, since they are typically difficult to standardize and reproduce [4-6]. A number of modified assays have been designed to overcome this problem, such as microfabrication printing [7], electrical impedance [4], and semi-automated press techniques [8], but have not reached widespread application. Another traditional cell migration assay, the Boyden chamber technique as variously modified [2], is widely used but its major limitations are that single cells cannot be visualized and collective cell migration is not testable. That is, these assays capture only the average behavior of a cell population, which can mask underlying dynamics and other valuable information about cell interactions (e.g., cell line heterogeneity, cell-ECM interface). Perhaps for these reasons, this technique has often yielded data inconsistent with *in vivo *findings [4,5]. Cell invasion assays based on three-dimensional (3-D) microscopy [9] provide excellent data collection at the single cell level, and track collective migration, but typically require several days or weeks of incubation for formation of colonies and use advanced microscopy methods for analysis, making them unsuitable for mid- and high- throughput studies. Further, migration assays designed for microplate readers or confocal microscopy typically require labeling of cells (e.g., using fluorescent probes) either prior to or after incubation – often an undesirable parameter [10]. In summary, many of the discussed techniques supply information about the average motility of a cell population, but fail to provide sufficient resolution for yielding precise information about individual cells or their spatial arrangement. Other techniques provide information on single cells and their arrangement, but are low-throughput. Together, the aforementioned techniques have provided important focused insights into cell motility mechanisms, as they are generally limited to measuring one parameter at a time [4,5], and their output is still adequate for many uses. However, we submit that there is an increasing need for a standardized, flexible, objective invasion assay with high-resolution for inspection of individual cells that can provide quantitative spatial information in a timely manner. This need is made more acute by the rise, in recent years, of theoretical Cancer Systems Biology approaches, in order to better incorporate the complex, multi-factorial interplay of tumor cells with their microenvironment [9].

**Figure 1:**
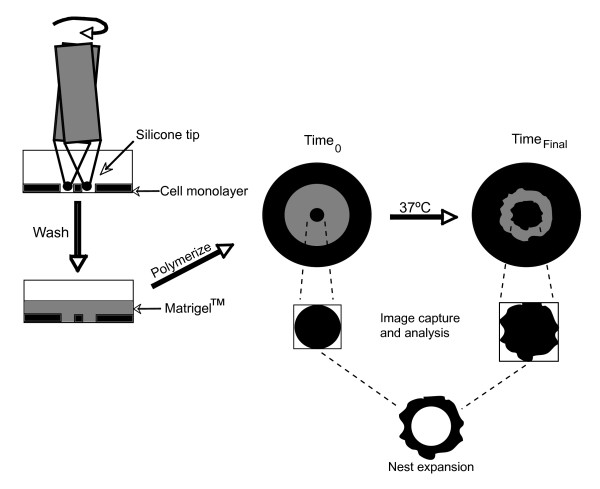
**Schematic of Nest Expansion Assay (NEA)**. Slightly altering our previous Circular Invasion Assay (CIA), the NEA was created by purposely tilting a sterile, flat-bottomed, silicone-tip fitted to a drill press, to leave an ~800 μm circular nest, or island of cells within the wounded area, in order to examine outward growth of cells, which mimics the directional spread of a tumor mass *in vivo*. Post wounding, Matrigel was laid and allowed to polymerize for 30 min, dishes were incubated at 37°C for indicated times, and nest expansion was calculated by comparing the area of nests at 0 h to the corresponding area at a time point of interest, using Java's *ImageJ*.

The NEA builds upon our previous Circular Invasion Assay (CIA; [11]). We now include, as a standard procedure, a Matrigel overlay, which is representative of tumor growth into surrounding tissues *in vivo *[12]. Several *in vitro *invasion studies have shown that inclusion of this component leads cells to exhibit closer behavior to that seen *in vivo *[3]. However, the key improvement is that a silicone-tipped drill press is used to create circular nests of cancer cells within an intact monolayer. Expansion of these nests is then recorded by high content microscopy (Figure 1). This experimental design overcomes the limitation of the CIA, as well as standard inward growth "scratch" assays, whose utility in defining the contour of an advancing cell margin is quickly extinguished once the wound is filled. In the NEA, the advancing cell margin is directed outward, providing more space and time for its examination. Since the NEA uses high-resolution microscopy and image analysis, this approach also enables a focus on the dynamic border regions of nests during the expansion process. Tumor expansion *in vivo *also occurs primarily at the tumor border [13], further justifying our emphasis on analyzing this nest region *in vitro *and determine its relationship to *in vivo *processes.

We also focused our efforts on efficiently and objectively quantifying the NEA experimental output. Straightforward "nest expansion" measurements (based on area) were systematically captured by computer-aided analysis of phase-contrast, time-lapse microscopy images using Java's freely available *ImageJ *software [14]. However, some irregular patterns, such as contours of biological cells or tumor colonies, are more difficult to describe using simple Euclidean measures (e.g., diameter, length); these objects can instead be quantitatively assessed using measures of complexity [15]. One such measurement that captures the irregularity of contours, or the borders of invasive nests in our case, is called the fractal dimension (D_f_) [16]. Fractal analysis is a tool sometimes employed in the fields of pathology and radiology to measure the irregularities associated with cancer growth and prognosis. In the past, it has been applied as a tool for assessing melanoma lesions *in situ *[17], glioblastoma invasion captured by MMR scanning [18], activated lymphocytes *in vitro *[19], and various cancer masses extracted from both laboratory animals and human patients [16,20,21]. Fractal interfaces between tumor and non-tumor regions (i.e., ECM) show temporal and spatial variances during the process of "roughening", or the increase of irregularity of a growth front, and can be used as an indicator of whether the tumor is likely to become infiltrative or not [16]. Ultimately, the real value of this measurement is that it provides an objective, quantitative approach for classifying organization and/or disorganization, something that is difficult for pathologists to do by eye [16]. To our advantage, some available software, such as *ImageJ'*s Fractal Dimension and Lacunarity plugin (*FracLac*; [22]), can assess images for this measurement with some user interaction and troubleshooting (and is freely available online). We therefore employed this quantitative technique to assess the advancing borders of nests in the NEA.

The NEA was designed with Cancer Systems Biology in mind, in that the spatial nature of its setup makes its outcome more susceptible to the simultaneous influence of many cellular parameters (e.g., motility, protease secretion, cell-cell adhesion, cell-matrix adhesion). These techniques are necessary for directly probing the complex interactions between cells and the microenvironment, particularly at the single-cell level, in order to reconstruct, e.g, with the aid of mathematics and computation, networks and mechanisms associated with cancer.

## Availability and requirements

### Cell Culture

MCF10A (and MCF10A-GFP), a human cell line derived from spontaneous immortalization of breast epithelial cells that is non-tumorigenic in nude mice [23], and MCF10A-CA1d (CA1d), a cell line derived from xenograph-passaging in nude mice creating a more aggressive, metastatic cell line [24], were maintained in constant culture. For a detailed description of method, see Additional File 1. Both cell lines are readily available through the Vanderbilt Integrative Cancer Biology Center's (VICBC) Tissue Culture Core Unit .

### Nest Expansion Assay (NEA)

Slightly altering our previously developed circular invasion assay (CIA; [11]), uniform, circular, artificial wounds were generated using a stabilized, rotating, silicone-tipped drill-press (Delta Shopmaster, Type 1, Model DP200). For the NEA, we purposely tilted the sterilized silicone tip to leave a circular nest of cells (8 per dish; ~800 μm in diameter) within each wounded area in order to examine outward cell invasion into overlaid Matrigel (Figure 1). For a detailed description of this method and its optimization, see Additional File 1.

### Live Cell Imaging

Time-lapse microscopy was conducted using a Zeiss Axiovert 200 M microscope (Zeiss, Thornwood, NY; 2.5× Plan NEOFLUR objective, NA 0.075; 10× Achroplan, NA 0.25, Ph1 objective) equipped with a Hamamatsu ORCA-ER CCD camera and temperature- and CO_2_-controlled chamber. Microscopy was under the control of OpenLab software (Improvision, Lexington, MA). At the beginning of each experiment (0 h), phase-contrast images of "wounded" monolayers were microscopically examined for standard reproducible cuts, images of each captured, and irregular outliers were discarded from the data set (negligible; data not shown). Nests expanding into the wounded areas were subsequently imaged at regular time points for up to 36 h.

### Image Processing and Nest Expansion Quantification

Preliminary image processing was performed (to isolate nest region) using Adobe Photoshop 7.0 (Adobe Systems, Inc., San Jose, CA) and "nest expansion" quantification obtained using Java's *ImageJ *software [14]. For a detailed description of these methods, see Additional File 1.

### Fractal Image Analysis

Images were further processed with Adobe Photoshop 7.0 (to obtain nest contours) for subsequent D_f _analysis via Java's *ImageJ *software with added *FracLac *plugin [22]. For a detailed description of these methods, see Additional File 1.

### Statistical Analysis

Statistical analyses were performed using SPSS, version 16 (SPSS Inc., Chicago, IL). Each cell line was sampled at least 8 times (N ≥ 8), for each treatment. To avoid confounding problems with multiple analyses along the time-response curve, final differences were only analyzed at 0, 10, 22, 28 and 36 h (as indicated). Differences between cell lines and treatments were examined using Student's t-tests (2-sided), and were considered significant when P < 0.05.

## Results and discussion

### NEA captures cell line invasiveness in vitro

**Figure 2:**
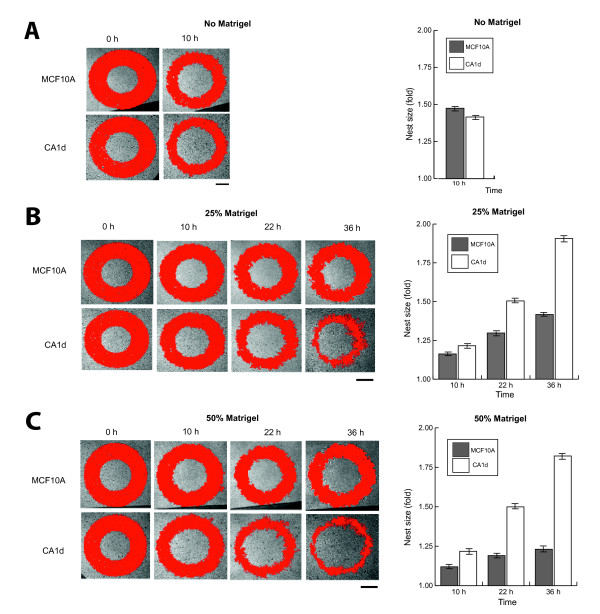
**NEA: Matrigel Overlay Differentiates Invasiveness *in vitro***. For this technique, artificial wounds were created with a silicone-tipped drill press to leave uniform, circular "nests" within a confluent monolayer of MCF10A or CA1d cells in Petri dishes, and Matrigel (25% or 50%) was added where indicated. Time-lapse images (0, 10, 22, 36 h) of expanding nests were obtained using a Zeiss Axiovert 200 M microscope equipped with a Hamamatsu ORCA-ER CCD camera (2.5×; scale bars = 500 μm). Nest expansion was calculated using *ImageJ *after applying thresholding and pseudo-color (red) functions to images. All values are presented as the mean ± standard deviation for each cell line, to reflect the fold increase in pixel number of each nest captured at each time point of interest, compared to the same nest at time point 0 h. (A) In the absence of the Matrigel overlay, the non-aggressive MCF10A cell line expanded significantly more than the aggressive, invasive CA1d cells after 10 h (N = 8; P = 0.03). However, time points 22 h and 36 h were immeasurable (results not shown) because all nests fully expanded into the outer ring of the remaining cell monolayer after this duration of incubation. (B/C) In contrast, in the presence of 25% or 50% Matrigel, CA1d nests expanded significantly more than MCF10A nests at all time points measured after 0 h (N ≥ 8; P < 0.05 in all cases). These results suggest that the ECM-like component is key to capturing cell lines' invasive potentials, at least for these cells.

The well-characterized cell lines, MCF10A and CA1d, have opposite degrees of invasiveness *in vivo *(non-aggressive, non-invasive, non-tumorigenic phenotype versus highly invasive, tumorigenic phenotype, respectively; [23,24]). To determine whether the NEA could produce results compatible with these *in vivo *findings, we first applied it to these cells both in the presence and absence of Matrigel for comparison. Multiple applications of a tilted, rotating silicone-tipped drill press on a confluent monolayer of cells in a Petri dish left behind uniform nests of cells (Figure 1). As shown in Figure 2, images were then systematically captured at each time point of interest (0–36 h), pseudo-color (shown in red) was applied to the "wound" rings to isolate regions of interest (ROI; i.e., nests) using a basic thresholding function in Adobe Photoshop, and nest areas (in pixels) were subsequently measured using *ImageJ*. Starting areas of nests (at 0 h) were found to be highly reproducible, with negligible intra-operator variance experienced (< 3.0%, results not shown).

*ImageJ *analysis revealed that, in the absence of Matrigel, MCF10A nests expanded somewhat more than CA1d nests after 10 h (Figure 2A; N = 8; P = 0.032). After 22 h and 36 h of incubation, nests of both cell lines fully expanded into the outer ring (results not shown). In contrast, in the presence of 25% or 50% Matrigel, CA1d nests exhibited significantly greater (N ≥ 8; P < 0.001 for all cases) levels of expansion than MCF10A nests, at all time points measured (Figure 2B and 2C). Further, nests in 50% Matrigel were smaller than nests in 25% Matrigel at all time points, particularly later ones. Taken together, these results suggest that the presence of an ECM-like overlay is a key ingredient in the NEA, in order to capture *in vivo *invasive properties. Further, the NEA uniform, reproducible nests, coupled with the *ImageJ*-based quantitation technique produced an effective and robust assay, as reflected by the small deviations of measurements for each group (Figure 2).

### Fractal analysis distinguishes noninvasive from invasive fronts

In the NEA, the invasive front of nests into the overlaid Matrigel barrier is examined by direct microscopic visualization. Obtaining quantitative spatial measurements at this cell-ECM interaction site, arguably the most important area of activity during tumor invasion [25], has proven to be a difficult feat by most classical methods [15]. However, fractal analysis has emerged as one approach to measuring the irregularity, or "complexity", of cell or colony borders. This tool can be an efficient and objective means for describing these complex shapes, otherwise subject to person-to-person variance.

**Figure 3:**
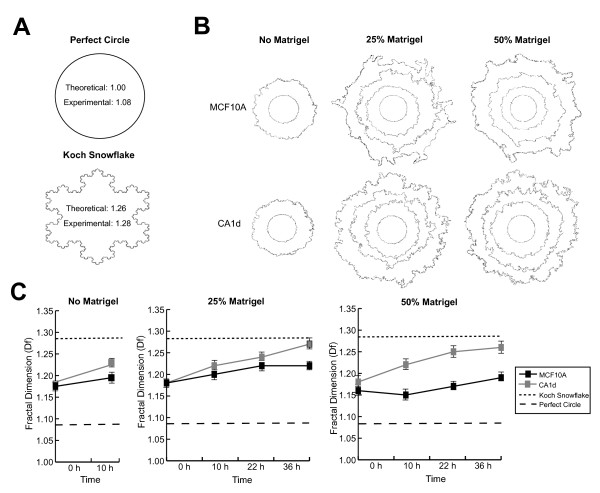
**Fractal Analysis of MCF10A and CA1d Cell Lines**. (A) Two simulated test patterns of known D_f_, a "perfect" 2-D circle and the "Koch snowflake", were used to validate the *FracLac *program, and to put our data into context within these controls. These patterns were normalized by pre-processing in the same manner as the contours of nests prior to fractal analysis, and resulted in measured values of 1.08 and 1.28, respectively. (B) Representative time-lapse images (0, 10, 22, 36 h) of MCF10A and CA1d cells taken in the absence or presence of 25% or 50% Matrigel overlay were thresholded to normalize background levels, and outlines were systematically applied to borders using a "define contours" function. D_f _measurements were then generated using the *FracLac *plugin. (C) All values are presented as the mean ± standard deviation of the fractal value for each nest captured at each time point of interest. Since NEA is highly standardized, all nests across both cell lines, and all treatments, had similar fractal measurements at 0 h. However, at all other time points measured after 0 h, the aggressive, invasive CA1d nests exhibited significantly greater D_f _measurements than the noninvasive MCF10A cell line (N = 8 per group; P < 0.01 in all cases), regardless of the microenvironment. Slopes calculated for lines fit to time-course data for CA1d D_f _values were approximately 2-fold greater than slopes for MCF10A nests, for all experimental conditions measured.

Java's *ImageJ FracLac *plugin assesses images for the D_f _measurement, providing that the user supplies the program with adequately processed images to isolate the ROI. We used this plugin to obtain D_f _measures for the nest contours, which outline the leading edge of expansion. We first explored and validated *FracLac *by analyzing two classical, simulated test patterns of known D_f_, a perfect two-dimensional (2-D) circle [26] and the "Koch snowflake" ([27]; Figure 3). Such mathematical fractals have constant D_f _across scales (i.e., displaying self-similarity), making such "controls" useful in optimizing settings, and putting other datasets into context. These patterns were pre-processed in the same manner as the nest contours prior to D_f _analysis, and resulted in measured values of 1.08 and 1.28 respectively, which are similar to the theoretical values (Figure 3A). Contours of MCF10A and CA1d nest borders from representative images are shown stacked in Figure 3B (0, 10, 22, 36 h; from inside out). MCF10A and CA1d cell lines exhibited varying degrees of D_f _or "complexity" over time (Figure 3C). Since the NEA is highly standardized and reproducible, nests across cell lines and treatments had similar D_f _measures at 0 h (N ≥ 8; P > 0.05, in all cases), which matches the finding that nest areas were also similar at this time. After 10 h of incubation in the absence of Matrigel, there was a significant difference in D_f _between cell lines (N = 8; P = 0.006). In the presence of either dilution of Matrigel, both cell lines exhibited a fairly step-wise increase of D_f _measures from 0–36 h. However, CA1d exhibited more irregular and invasive borders leading to significantly greater (N = 8; P < 0.01, in all cases after 0 h) D_f _measurements than MCF10A nests, which displayed smooth, less protruding borders. Slopes calculated for lines fit to time-course data for CA1d D_f _values were approximately 2-fold greater than those slopes for MCF10A nests in all instances. These results suggest that CA1d nests exhibit more "complex" borders than MCF10A nests. Further, both cell lines exhibited increasingly "complex" borders over time, with the greatest measurements occurring at 36 h, which corresponds with the idea that tumors typically gain "irregularity" over time, perhaps as part of the invasion process. These D_f _results, like nest expansion measurements, confirm that the NEA and image analysis techniques can capture differences between the front of noninvasive and invasive cells, at least for cell lines with widely diverging invading behavior.

### NEA and fractal analysis capture invasive differences linked to microenvironmental conditions

A few previous studies have reported invasive "fingering" patterns at the edge of certain types of tumors both *in vitro *and *in vivo*, which depend on microenvironmental conditions [28,29]. A few mathematical and computational modeling approaches have also demonstrated this microenvironment-dependent pattern in *in silico *tumors [30]. One such model, the Hybrid Discrete-Continuum (HDC) mathematical model presented in Anderson *et al. *[30], reported an association between stressful conditions and invasive front complexity. In the HDC model, a 2-D lattice represents the tissue domain where cells reside, including ECM and other factors [30]. Using this approach, the model can predict various patterns of invasion dependent upon cells' interactions with their microenvironment (as represented by various parameters in model).

To test ideas generated by HDC *in silico *results, we slightly modified the initial NEA method to examine co-cultured, fluorescently labeled cells under two different microenvironmental conditions. Specifically, MCF10A cells were GFP-labeled prior to seeding (MCF10A-GFP), and final nests were fixed and stained with rhodamine-phalloidin to mark actin filaments, in order to visualize unlabeled cells (CA1d) prior to fluorescence imaging. End-point assays were performed with MCF10A-GFP or CA1d alone, or with MCF10A-GFP mixed with either unlabeled MCF10A (1:1) or CA1d (1:1). All nests were overlaid with a single, 50% Matrigel density, to model space constraints. Nests were allowed to expand in the presence or absence of serum for either 36 or 28 h, respectively. The shorter incubation period was required for serum-free conditions, because cell death became an issue with longer times. Microscopic images were then processed and assessed for both nest expansion and Df measures. Note that cells on the other side of outer "wound" rings were excluded from analysis.

**Figure 4:**
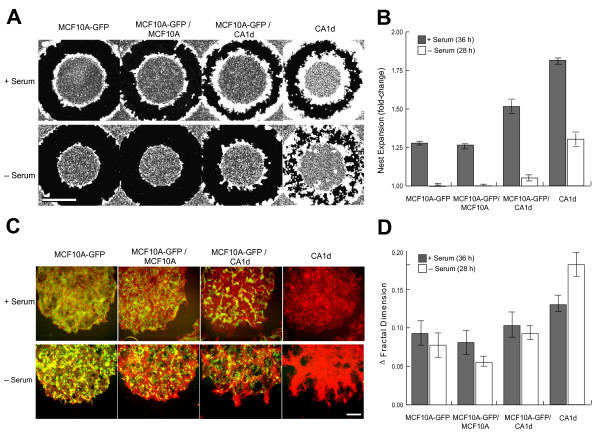
**Fractal Analysis of Mixed Culture MCF10A and CA1d Cell Lines**. (A) Nests were created with MCF10A-GFP alone, MCF10A-GFP mixed with either unlabeled MCF10A or CA1d cells (1:1), and with CA1d alone. Nests were then overlaid with 50% Matrigel and allowed to expand in either the presence or absence of serum for 36 or 28 h, respectively. Scale bar is 1000 μm. (B) In the presence of serum, CA1d nests expanded significantly more than MCF10A after 36 h (N = 8; P < 0.001, in all cases), and 1:1 MCF10A+CA1d colonies expanded at an intermediate rate. Similar results were observed in the absence of serum. (C) Magnified images (10×) of nests with fluorescent labels for visualization of individual cell types (pre-labeled MCF10A-GFP (green); final nests were fixed/stained with rhodamine-phalloidin to mark actin filaments and visualize CA1d cells (red). Importantly, in the mixtures of MCF10A-GFP and CA1d in normal culture conditions (+ serum), there is clear trapping of groups of MCF10A cells in the inner portion of the nests by expanding CA1d cells (upper panel). Serum deprivation in the space-constrained environment gives a similar result, except that the difference between CA1d and MCF10A-GFP expansion is amplified (lower panel). Scale bar is 600 μm. (D) Fractal dimension measures confirm the differences observed at nest borders across MCF10A and CA1d cells in the two conditions tested. Data is presented to include only the change in D_f _from 0 h to the final end point measured. In normal tissue culture conditions, MCF10A-GFP, MCF10A-GFP+ MCF10A (1:1), and MCF10A-GFP+CA1d (1:1) nests displayed comparable changes in D_f _measures from 0 h to 36 h (N = 8; P > 0.05, in all cases). CA1d cells alone led to somewhat larger measures than other nest types. In serum-deprived conditions, the separation between MCF10A and CA1d nests increased dramatically. MCF10A-GFP, MCF10A-GFP+MCF10A (1:1), and MCF10A-GFP+CA1d (1:1) nests again displayed comparable D_f _measures from 0 h to 28 h (N = 8; P > 0.05, in all cases). However, in the absence of serum, CA1d nests had drastically larger measures than all other nest types (N = 8; P < 0.001, in all cases).

Figure 4A includes representative low-magnification (2.5×) composite images of final nests superimposed on 0 h nests, revealing clear differences in nest expansion (shown in white) across the various cell mixtures and conditions. In the presence of serum, CA1d nests expanded significantly more than MCF10A-GPF and MCF10A-GFP:MCF10A nests (Figure 4B; N = 8; P < 0.0001), and MCF10A-GFP:CA1d co-cultures (1:1) expanded at an intermediate rate (Figure 4B; N = 8; P < 0.001, in all cases). In the absence of serum, the same trend was observed, but expansion was drastically reduced for all nest types. In the MCF10A-GFP:CA1d mixtures, MCF10A cells appeared to be trapped in the inner portion of the nests, more so in serum deprived conditions (Figure 4C), which could relate to a combination of factors, including cell-cell adhesion, motility or rate of proliferation. Any or all of these factors could be deconvoluted by further high-content microscopy analyses, supporting the global outlook on invasion gained with the NEA.

In magnified images (10×; Figure 4C), nest margin contours are better appreciated. D_f _measurements (from 2.5× images) indicated that the nest margin complexity was similar across cell lines and conditions at 0 h (data not shown). However, at the end point, D_f _differed markedly between MCF10A-GFP alone and both of the co-cultures (N ≥ 5; P > 0.05, in all cases), while CA1d cells alone led to larger D_f _measures than all other nest types (Figure 4D). In the absence of serum, separation between MCF10A and CA1d nests increased dramatically. That is, MCF10A nests had similar D_f _measures, MCF10A-GFP:CA1d had intermediate measures, and CA1d nests had drastically larger measures than all other nest types (Figure 4D; N = 8; P < 0.001, in all cases).

One of the major predictions of the HDC model is that under stressful conditions of growth and space constraints, more aggressive phenotypes become dominant [30]. The experimental observation that MCF10A cells were trapped by the aggressive CA1d cells in the mixed nests of the NEA agrees with that prediction. Furthermore, the HDC model predicted more complex margins in colonies of aggressive cells, under stressful conditions [30]. The NEA finding also agrees with this prediction. Clearly, these initial correlations show that there is merit to the NEA, but additional in-depth studies are needed to solidify these tentative conclusions.

In summary, the benefits of the NEA approach are many. For instance, because we use a machine-based approach (drill press to create wounds, and computer-assisted analyses for measurements), the assay setup is not subject to operator variance, and is both highly reproducible and objective. Nonetheless, the NEA setup is flexible to introduction of various perturbations (e.g., additional and diverse microenvironmental stressors). Since nests can be assessed for area and D_f _simultaneously, a more detailed quantitative picture of cells' invasive potential is achieved with a single assay. Lastly, because NEA relies on high-content microscopy imaging, cells are examined both at the population and the single-cell level, making it particularly useful for individual-based mathematical/computational modeling. We are hopeful that this tool will help bridge the gap between *in silico *outcomes and *in vivo *validation.

## Abbreviations used

2-D: two-dimensional; 3-D: three-dimensional; CIA: Circular Invasion Assay; D_f_: fractal dimension; ECM: extracellular matrix; HDC: Hybrid-Discrete Continuum; NEA: Nest Expansion Assay; ROI: region of interest.

## Competing interests

The authors declare that they have no competing interests.

## Authors' contributions

YK performed all NEA experiments, contributed intellectual property, and contributed to manuscript preparation; AK performed fractal analyses and contributed to manuscript preparation; BW performed data analysis, statistical testing, and contributed to manuscript preparation; LE contributed to manuscript preparation; ARA contributed to manuscript preparation; VQ conceived of this study and contributed to manuscript preparation.

## Supplementary Material

Additional file 1 **Supplementary text.** Click here for file
