# Supplementary material for: Nest expansion assay: a cancer systems biology approach to in vitro invasion measurements
Source: BMC Res Notes. 2009 Jul 13;2:130. doi: 10.1186/1756-0500-2-130 (PMC2716356; doi:10.1186/1756-0500-2-130)
Supplement: Additional file 1 — Supplementary text. [file 1756-0500-2-130-S1.doc]

**Supplemental Text**

## Cell Culture

MCF10A (and MCF10A-GFP), a human cell line derived from spontaneous immortalization of breast epithelial cells that is non-tumorigenic in nude mice (23), and MCF10A-CA1d (CA1d), a cell line derived from xenograph-passaging in nude mice creating a more aggressive, metastatic cell line (24), were maintained in GIBCO® DMEM/F-12 media (Invitrogen, Carlsbad, CA) supplemented with 5% horse serum (Invitrogen), 0.1 µg/ml cholera toxin (Calbiochem/EMD Biosciences, Gibbstown, NJ), 10 µg/ml insulin (Invitrogen), 0.5 µg/ml hydrocortisone (Sigma, St. Louis, MO), and 20 ng/ml epidermal growth factor (Invitrogen). Cells were kept in constant culture in a humidified atmosphere of 5% CO_2_ at 37°C. Cells (1.5 x 10^6^) were seeded, in sterile conditions, on polysterene, 35-mm, tissue culture-treated Petri dishes (Corning, Lowell, MA) for ~18-24 h.

*Nest Expansion Assay (NEA)*

Slightly altering our previously developed circular invasion assay (CIA; 11), uniform, circular, artificial wounds were generated using a stabilized, rotating, silicone-tipped drill-press (Delta Shopmaster, Type 1, Model DP200). For the NEA, we purposely tilted the sterilized silicone tip to leave a circular nest of cells (8 per dish; ~800 µm in diameter) within each wounded area in order to examine outward growth, which more closely mimics the directional spread of a tumor mass *in vivo* (Figure 1). The optimal size and shape of wounds, their spacing and number, and various other parameters were established in preliminary experiments (results not shown). The silicone tip (manually cut down to a rounded, 1 mm-diameter shaped tip, with a flat bottom using a razor blade) was regularly washed with 70% ethanol between preparations of individual dishes. Cell debris created by excision of cells was removed from each dish by manual pipetting, and intact cells were gently washed with PBS twice. Where indicated, 25% or 50% Matrix Growth Factor Reduced Matrigel (BD Biosciences, San Jose, CA; in DMEM; 600 µl total volume) was overlaid in each dish and allowed to polymerize for at least 30 min. The main components of this reagent are laminin-1, collagen IV, heparin sulfate proteoglycans, entactin, and various other growth factors and proteases, all of which comprise vertebrate basement membranes *in vivo*. Two ml of growth media was added to each dish. Dishes were then incubated in a humidified atmosphere of 5% CO_2_ at 37°C for up to 36 h. Where indicated in mixed culture experiments, nests were fixed and stained for F-actin with AlexaFluor 568 (Invitrogen). The optimal concentrations/volumes of all reagents were determined in previously performed dose-dependent experiments, taking concentrations given in the literature as baseline values (results not shown).

## Image Analysis and Nest Expansion Quantification

Using Adobe Photoshop 7.0 (Adobe Systems, Inc., San Jose, CA), nests were distinguished from the surrounding, cell-free wounded areas by applying an automatic, software-defined threshold to each image (to remove both cell-free areas and intact cell regions not of interest from quantitation), and pseudo-color applied to these areas. Converted, time-lapse images containing nests were quantified using Java’s *ImageJ* software (14), by auto-selecting each nest using the “wand” tool, and capturing the area measurement. Appropriate pairs of corresponding images were overlaid and compared to one another, to determine the difference between nest areas measured from the original time point (0 h) to the final time point of interest (10, 22, 28, or 36 h). This difference (in pixels) was then calculated and presented in terms of the fold change of “nest expansion” for each wounded area. All values are presented as the mean fold-change ± standard deviation for each cell line.

## Fractal Image Analysis

Using Adobe Photoshop 7.0, phase-contrast images were further processed to focus in on each nest by manually selecting each region of interest (i.e., nests) and using the basic “crop image” function. Magnified images were then processed by applying a “binary contours” function, in order to define an outline of each nest’s borders and to exclude all other pixels. Images of nest contours were finally assessed for fractal dimension (D_f_), or a measure of “complexity”, using Java’s *ImageJ* software with added *FracLac* plugin *(*22). Briefly, *Fraclac* lays a series of grids of decreasing box sizes over an image, and for each grid, records the number of boxes that fall on the image and the number of pixels per box for each box size. From this data, *Fraclac* derives the D_f_ measurement, which is the slope of the least squares fit linear regression line from the log-log plot and epsilon (box size/maximum image dimension) on the x-axis and count on the y-axis. *FracLac* scans an image multiple times to minimize bias associated with the location of the scanning grid. That is, the algorithm finds an average D_f_ measurement over all scans, as well as a "most efficient" D_f_ measurement using these scans. The data depend on the position of the grid over the image. To minimize grid-associated bias, *FracLac* can calculate several measurements over different grid positions of a single image. The algorithm selects the position of the top left corner of the grid from within a range of the top left corner of the smallest rectangle enclosing the pixelated area. Other than this first origin (the top left corner), the other origins are selected randomly, so that different information is read each time the image is scanned randomly, so that different information is read each time the image is scanned.

For our analyses, *FracLac* used the box counting dimension from a 1-origin count with no smoothing filter, a minimum box size of 2 pixels, and a maximum box size of 45% of the pixilated part of each image (all user-selected options; can be adjusted for different data sets), which output the D_f_ of each nest border region. Appropriate pairs of contoured images were compared to one another, to determine the difference in complexity (i.e., D_f_ measurements) between border regions that were measured from each original time point (0 h) and the appropriate final time point of interest (10, 22, 28, or 36 h). All D_f_ values are presented as the mean ± standard deviation for each cell line.
